# Health Behavior and Associated Factors in Young Adult Cancer Patients

**DOI:** 10.3389/fpsyg.2021.697096

**Published:** 2021-09-01

**Authors:** Isabelle Stroske, Kristina Geue, Michael Friedrich, Annekathrin Sender, Ricarda Schmidt, Diana Richter, Katja Leuteritz

**Affiliations:** ^1^Department of Medical Psychology and Medical Sociology, University Medical Center Leipzig, Leipzig, Germany; ^2^Clinic and Polyclinic for Psychosomatic Medicine and Psychotherapy, University Medical Center Leipzig, Leipzig, Germany

**Keywords:** cancer, young, lifestyle, adolescent and young adults, health behavior

## Abstract

**Objectives:** Having cancer in young adulthood increases the risk of adverse long-term health effects. These risks can be influenced by one’s health behavior (HB). The aim of this study is therefore to investigate the presence of health behavior in adolescents and young adults (AYAs) and to identify associated factors.

**Design:** Young cancer patients (18–39 years old at time of diagnosis) were surveyed at baseline and 12 months later via online or as a paper-pencil version.

**Methods:** A spectrum of indicators for HB was assessed via seven items from the Questionnaire of Multiple Health Behavior (MHB). Multiple linear regression analyses were conducted to determine factors associated with patients’ HB indicators.

**Results:** Five-hundred and fourteen AYAs (75% women) reported the highest level of health-conscious behavior for “avoidance of consumption of nicotine,” “follow medical recommendations,” and “being considerate in road traffic.” Less health-conscious behavior was reported for “keeping an appropriate and balanced diet” and “physical activity.” Significant improvements from baseline to the follow-up were observed for “regularly attending health screening” (Hedges’ g = 0.44). The analyzed factors explained up to 10% of the HB indicators. Women reported significantly more health-conscious behavior than men in four out of seven HB indicators. Higher quality of life (QoL) was associated with more health behavior in three out of seven HB indicators.

**Conclusion:** Findings show a predominantly health-conscious lifestyle in AYA cancer patients, though we also found harmful behavior which needs to be better approached—e.g., through improving AYAs’ health education. AYA men should be particularly targeted in specific prevention and health promotion measures. Future work should identify other factors associated with HB to evaluate targets for intervention.

## Introduction

Worldwide, around one million adolescents and young adults (AYA) are diagnosed with cancer each year (Bleyer et al., 2017). Over the last decades, the overall incidence rate of cancer in this age group has not changed much (<1% increase per year) in Europe nor in the United States or Canada (Lewis et al., 2014; Smith et al., 2016; Fidler et al., 2017). The National Cancer Institute defines the widest age range of AYAs from 15 to 39 years of age at the time of diagnosis (National Institutes of Health, 2021). However, medical forecasts for AYAs are well above average: the 10-year overall survival rate for 20–39 year olds is about 80% (Zebrack, 2011; Kaul et al., 2017). Although the number of AYA long-term survivors is steadily increasing, AYA survivors have an increased risk of adverse long-term health effects like cardiovascular diseases or secondary neoplasms (Fosså et al., 2004; Hilgendorf et al., 2016; National Institutes of Health, 2021). Notably, cancer survivors’ risk of secondary diseases and cancer recurrence can be influenced by their own health behavior (Klosky et al., 2007; Fairley et al., 2009; Li et al., 2009; Schüz et al., 2015).

Health behavior describes all behaviors or activities of people that have been shown to increase the probability that health will be maintained, promoted, or improved, or that disease will be avoided or detected early (Kaptein and Weinman, 2004; Faltermaier, 2015). According to Schwarzer (2004), health behavior includes not only health-promoting behaviors (such as physical activity and healthy nutrition) but also the avoidance of risk behaviors (such as legal and illegal substance use). Empirical work highlights the multidimensionality of the health behavior construct (Schwarzer, 2004; Sallis et al., 2008; Havigerová et al., 2019). With an overall healthy lifestyle, realized through a combination of multiple healthy behaviors, lower disease and mortality rates have been empirically proven among the general population (Loef and Walach, 2012; Lange and Finger, 2017). There is no complete conformity with regard to the type and number of dimensions with which health behavior can be assessed. But there is agreement that physical activity, balanced nutrition, substance avoidance, preventive health care, avoidance of environmental risks, and stress have been found to be relevant for health behavior in the healthy general population as these factors are among the most important determinants of non-communicable chronic diseases (Kulbok and Cox, 2002; Lange and Finger, 2017). For AYA cancer survivors, (preventive) health check-ups and following medical advice are particularly important to reduce the risk of secondary diseases and cancer recurrence (Butow et al., 2010; Rosenberg et al., 2013).

Many patients are in poor physical or cognitive general condition after a cancer diagnosis and its treatment. Radiation, cytostatics, analgesics, or anesthetics can also lead to fatigue and restrictions in the ability to drive (Yuen et al., 2008; Mailis-Gagnon et al., 2012). At the same time, young cancer patients are mobile due to their age and the need for medical treatment and follow-up appointments. Therefore, we consider behavior in road traffic as another relevant aspect of AYA cancer patients’ health behaviors.

Although AYAs have been studied extensively over the past decade, thereby providing evidence for high psychological distress, especially anxiety (Geue et al., 2019), and worse quality of life (QoL; Quinn et al., 2015), health behavior, as an essential preventive factor, has rarely been examined so far (Warner et al., 2016). There are initial indications in AYAs that more pronounced fatigue is associated with less physical activity (Spathis et al., 2017) and that more pain is associated with higher alcohol and nicotine consumption (Milam et al., 2021). Four factors have been identified as risk factors for an unhealthy lifestyle in the general population: male gender, low income, younger age, and increased psychological distress (Allgöwer et al., 2001; Berrigan et al., 2003).

The review by Rabin and Politi (2010) showed that young adult cancer survivors do not sufficiently meet the general scientific recommendations regarding nicotine consumption, physical activity, and a healthy diet. Similarly, observational studies have shown increased nicotine consumption and a stronger expression of risky alcohol consumption among AYAs compared to their healthy peers (Coups and Ostroff, 2005; Tai et al., 2012; Warner et al., 2016). In the AYA population, high psychological distress has been found to be associated with smoking, lower levels of physical activity, and low fruit/vegetable intake; no association could be found with binge drinking (Warner et al., 2016). In addition, Masiero et al. (2016) highlight that especially for young cancer survivors, the combination of constant fear of disease recurrence and the need to develop self-identity supports engaging in harmful health behaviors like smoking.

Thus, psychological distress seems to be an important factor influencing health behavior in AYAs and the general population, and the influence of distress needs to be analyzed for more different health behavior dimensions. In general, existing studies on health behaviors in AYAs are mostly cross-sectional in nature and investigate selected health behaviors, such as alcohol consumption or nutrition, although health behavior comprises more than that.

Demark-Wahnefried et al. (2005) emphasized that after receiving a cancer diagnosis, patients are in a good position to learn new and healthy behavioral patterns and that they are more likely to change their nutrition, try to be more active, and quit smoking. It is a challenge to use this potential within the psychosocial care of AYA patients, but it is essential to do so because young adults are passing through a phase of life in which they develop lifelong habits (Daniel et al., 2015).

Thus, the presented exploratory study sought to investigate a spectrum of health behavior indicators and potentially associated factors (sociodemographic, medical, and/or psychosocial) in young German cancer patients at two time points. As there is little research on health behavior in the AYA group, our approach is exploratory and we do not formulate targeted hypotheses. Specifically, this study aimed:

• To determine degrees of health behavior of AYA cancer patients.
• To describe temporal changes in these health behavior dimensions over 12 months.
• To describe group differences between low- and highly distressed AYA patients in terms of health behaviors.
• To identify associated sociodemographic (e.g., gender), medical (e.g., cancer diagnosis), and/or psychosocial (e.g., QoL) factors for multiple health behaviors.

## Materials and Methods

### Design

The present data were derived from the prospective longitudinal AYA-LE study investigating the psychosocial life situation and psychosocial care in AYAs at two time points. For the AYA-LE study, participants had to fulfill the following inclusion criteria: (1) age at diagnosis: 18–39 years; (2) first manifestation of cancer (all malignant tumor entities); and (3) diagnosis must not be longer than 4 years ago. The baseline survey was conducted between May 2014 and December 2015. The 12-month follow-up was finished in December 2016. The study obtained ethical approval from the ethics committee (Ref.-Nr. 372-13-16122013) of the medical faculty of the University of Leipzig, Germany.

Recruitment of participants took place nationwide in Germany by cooperation with 16 oncological acute care hospitals, four (cancer) rehabilitation centers, and two cancer registries. Participants meeting the inclusion criteria were informed about the study and invited to participate. After giving written informed consent for study participation, participants were sent a link to answer the standardized study questionnaire online with (Limesurvey GmbH, 2021) or, if desired, postally as a paper-pencil questionnaire. Participants received financial compensation of 10€ at each time point. More detailed information about the AYA-LE study has been given by Leuteritz et al. (2017, 2018).

### Measures

In addition to sociodemographic (gender) and medical data (age at diagnosis), self-report questionnaires on health behaviors, general psychopathology, pain, fatigue, QoL, and distress were administered.

The Questionnaire of Multiple Health Behavior (MHB) (Wiesmann et al., 2003) is a 39-item German self-report questionnaire on habitual health-related behavior. The MHB is based theoretically on the assumption that health behavior is a multidimensional construct (Hevey et al., 1998; Kulbok and Cox, 2002). The MHB includes six dimensions: active lifestyle, nutrition, substance avoidance, compliance, safety orientation, and hygiene, although the questionnaire is to be used as a total score (Cronbach’s alpha = 0.88). From these dimensions, we selected a short list of seven items for this explorative study. For the item-selection process, it was crucial that: (i) the selected MHB item had high content-relevance to cancer patients health (in terms of long-term health effects and secondary neoplasms) according to previous research literature; and (ii) had relevance to the group of young adolescent patients with cancer, if literature was available.

The seven items were rated on a five-point Likert-type scale (0—never, 1—rarely, 2—often, 3—almost always, and 4—always), wherein there are positive and negative formulated items. Negative formulated items are not inverted in the analyses. Instead of analyzing a score, the analysis was carried out at item level, because our selection of items is not validated as a questionnaire with one or more domains, and in order to be able to draw differentiated conclusions regarding health behavior.

Psychological distress was measured using the Hospital Anxiety and Depression Scale (HADS), a validated screening instrument for anxiety and depression (Herrmann et al., 1995; Herrmann-Lingen et al., 2011). It consists of 14 items rated on a four-point Likert scale (range from 0 to 3), constituting two summed subscales of anxiety and depression (Herrmann-Lingen et al., 2011). Higher values indicate higher levels of psychological distress. To differentiate between patients with different degrees regarding anxiety or depression, the original test authors defined the following ranges: 0–7 = non-cases, 8–10 = borderline cases, and 11–21 = cases (Herrmann et al., 1995). To distinguish between low- and highly distressed patients, patients exceeding the cut-off of ≥ 11 on at least one subscale (anxiety or depression) were classified as highly distressed. Patients with cut-off of < 11 on the subscale anxiety or depression were classified as low distressed. A literature review reported an average reliability (measured with Cronbach’s alpha) above 0.80 for the anxiety and for the depression scale (Bjelland et al., 2002). In our analyses, Cronbach’s alpha for the anxiety scale was 0.82 (t1) and 0.84 (t2). For the depression scale, Cronbach’s alpha was 0.82 (t1) and 0.85 (t2).

The European Organization for the Research and Treatment of Cancer Quality of Life Questionnaire-Core 30 (EORTC QLQ-C30) was used to measure patients’ QoL on five functioning scales, three symptom scales, six single-item symptom scales, and a two-item global health status/QoL scale (Aaronson et al., 1993). The EORTC QLQ–C30 comprises 28 items, which are scored on a four-point Likert scale (range from 1 to 4) and two items for the QoL scale which are scored on a seven-point Likert scale (range from 1 to 7). All scale scores are standardized to a range between 0 and 100 with higher values indicating better QoL, better functioning, or higher symptom burden. Acceptable values for a high reliability (α > 0.70) and good construct validity have been demonstrated for all scales of the German version (Schumacher et al., 2003). In our study, we used the following scales with the following values of Cronbach’s alpha: fatigue (three items, t1: 0.85, t2: 0.84), pain (two items, t1: 0.87, t2: 0.87), QoL (two items, t1: 0.87, t2: 0.88), and financial difficulties (single-item scale).

### Data Analysis

The statistical data analyses were performed by using the software package IBM SPSS Statistics 24 (RRID:SCR_019096) and Microsoft Excel 2010 (RRID:SCR_016137).

Means and standard deviations were calculated. Differences between t1 and t2 health behavior items were tested using the Student *t*-test for dependent samples. Mean differences between low- and highly distressed patients at t2 were tested using Student *t*-tests for independent groups. To judge the magnitude of effects, the standardized mean difference Hedges’ g was used, which is a bias-corrected version of the effect size Cohen’s d (Borenstein, 2009). Effect sizes can be classified as small (Hedges’ g ≥ 0.2), medium (Hedges’ g ≥ 0.5), or high (Hedges’ g ≥ 0.8) (Cohen, 1988).

Multiple linear regression analyses were conducted for seven indictors of health behavior from the MHB (dependent variables) at follow-up (t2), using the method “enter,” that is, all independent variables were included in the respective model at once. We included the following independent variables in each model: Variables to represent sociodemographic characteristics were gender (0 = male, 1 = female). For medical characteristics, we selected age at diagnosis (in years), pain (symptom item of EORTC QLQ-C30, range 0–100), and fatigue (three-item symptom scale of EORTC QLQ-C30, range 0–100, higher values represent higher fatigue). To represent psychological characteristics, we selected global health status (two-item QoL scale of EORTC QLQ-C30, range 0–100, higher values represent higher quality), distress (based on HADS, 0 = both subscales < 11, 1 = at least one subscale = 11), and cancer-related financial difficulties (symptom item of EORTC QLQ-C30, range 0–100, higher values represent more difficulties). The selection of these independent variables was based on previous literature that we have described previously (Allgöwer et al., 2001; Berrigan et al., 2003). Continuous independent variables were z-standardized (mean = 0, standard deviation = 1).

The largest correlation in terms of amount within the independent variables is between QoL and fatigue with | *r*| = −0.68, hence no correlation is above | *r*| > 0.8 and it can be assumed that there is no multicollinearity. Means, standard deviations, and correlations between independent variables are presented in Supplementary Table S1. For each model, we present the change in explained variance (Δ*R*^2^) after including the independent variables, as well as the corresponding F-value (ΔF) with degrees of freedom (df), and *p*-value. To compare the magnitude of effects between models, we also present Cohen’s f^2^ (Selya et al., 2012). Values in f^2^ ≥ 0.02 indicate small effects, ≥0.15 medium, and ≥0.35 large effects (Cohen, 1988).

The expectation–maximization algorithm was used to estimate the missing values for the MHB, HADS, PACIS, and EORTC QLQ-C30 variables (Dempster et al., 1977). Missing values have been estimated for baseline and follow-up at once including all *n* = 514 patients who participated at both time points. Additionally, we included the full information from age at diagnosis, gender, and time since diagnosis. Estimated missing values, which exceeded the possible range, were set to the nearest integer. Altogether we estimated missing values for 104 items of MHB (2 time points × 7 items), HADS (2 × 14 items), PACIS (2 × 1 item), and EORTC QLQ-30 (2 × 30 items). Overall 256 values (<1%) from a total of 104 items × 514 cases = 53,456 values were imputed.

## Results

### Sample

In total, 762 participants provided written informed consent to participation. Of these respondents, 185 were excluded (*n* = 43 withdrew their written consent, *n* = 88 did not meet the inclusion criteria, and *n* = 54 did not complete the questionnaire). Of the 577 participants who completed the first survey, 63 (11%) dropped out at the second survey. Table 1 summarizes the characteristics of the 514 patients completing both assessments. Patients’ average age at diagnosis was 29.6 years (SD = 6.1 years), and 386 patients (75.1%) were female. The most frequent cancer diagnoses were breast cancer (*n* = 139; 27.0%) and Hodgkin lymphoma (*n* = 93; 18.1%). At baseline, 309 patients (60.1%) specified a high school degree as their highest educational degree.

**TABLE 1:** Characteristics of the sample at baseline (*n* = 514).

	***N* (%)**
**Sociodemographic variables**	
Gender (female)	386 (75.1)
Age at diagnosis [Mean (SD)]	29.6 (6.1)
18–25 years emerging adulthood	158 (30.7)
26–39 years young adulthood	356 (69.3)
Partnership (Yes)^a^	363 (70.6)
Highest educational degree^b^	
No educational degree	5 (1.0)
Basic educational degree (<10 years)	30 (5.8)
Secondary educational degree (10 years)	165 (32.1)
High school degree (>10 years)	309 (60.1)
Children (Yes)	176 (34.2)
Housing/living conditions	
Single	123 (23.9)
With partner	296 (57.6)
With parents	41 (8.0)
Shared community	54 (10.5)
**Medical variables**	
Cancer diagnosis	
Breast cancer	139 (27.0)
Hodgkin lymphoma	93 (18.1)
Gynecological cancer	46 (8.9)
Testicular cancer	42 (8.2)
Hematological cancer	36 (7.0)
Non-Hodgkin lymphoma	32 (6.2)
Thyroid cancer	29 (5.6)
Gastrointestinal cancer	24 (4.7)
Sarcoma	21 (4.1)
Melanoma	17 (3.3)
Others	35 (6.8)
Time since diagnosis in months (at t1) Mean (SD), Min–Max	12.1 (8.1), 1.0–44.4
Medical therapy (multiple answers possible)	
Chemotherapy^c^	392 (76.3)
Radiotherapy^d^	242 (47.1)
Surgery	379 (73.7)
Transplantation^e^	30 (5.8)

*^a^Missing: 3 (0.6%);*

*^b^Missing: 5 (1.0%);*

*^c^Including radio-chemotherapy;*

*^d^Including radio-chemotherapy and nuclear therapy;*

*^e^Including bone marrow transplantation or stem cell transplantation.*

### Health Behavior Indicators and Differences Between Measurement Points

Mean levels of the health behavior indicators are displayed in Table 2. Health behaviors that were most commonly reported at t1 were “following medical orders” (M = 3.39; SD = 0.76), “consumption of nicotine” (inverse item: M = 0.47; SD = 1.06), and “being considerate in road traffic” (M = 3.03; SD = 1.09). For physical activity (M = 2.40; SD = 1.09) and “keeping an appropriate and balanced diet” (M = 2.57; SD = 0.93), the patients reported less health-conscious behavior. More than 20% of patients reported never being physically active.

**TABLE 2:** Differences of health behavior indicators between measurement points in AYA patients (*n* = 514).

		**T1**	**T2**	**Difference (T2−T1) Hedges‘ g (*p*)**
Regular health screening (e.g., medical check-ups and functional testing of organs)	Mean (SD) Never N (%) Frequently N (%) Always N (%)	2.78 (1.22) 94 (18.3) 121 (23.5) 299 (58.2)	3.28 (1.07) 48 (9.3) 79 (15.4) 387 (75.3)	**0.44 (*p* < 0.001)**
Following medical orders (e.g., medication intake)	Mean (SD) Never N (%) Frequently N (%) Always N (%)	3.39 (0.76) 10 (1.9) 54 (10.5) 450 (87.5)	3.45 (0.80) 13 (2.5) 48 (9.3) 453 (88.1)	0.08 (*p* = 0.090)
Keeping an appropriate and balanced diet (rich in vitamins, minerals, and fiber)*	Mean (SD) Never N (%) Frequently N (%) Always N (%)	2.57 (0.93) 57 (11.1) 184 (35.8) 273 (53.1)	2.52 (0.92) 64 (12.5) 192 (37.4) 258 (50.2)	−0.05 (*p* = 0.212)
Consumption of nicotine* (smoking cigarettes, cigars, pipe)	Mean (SD) Never N (%) Frequently N (%) Always N (%)	0.47 (1.06) 449 (87.4) 28 (5.4) 37 (7.2)	0.53 (1.14) 445 (86.6) 20 (3.9) 49 (9.5)	0.05 (*p* = 0.060)
Consumption of “harder” alcoholic beverages* (liquor, etc.)	Mean (SD) Never N (%) Frequently N (%) Always N (%)	0.54 (0.62) 494 (96.1) 16 (3.1) 4 (0.8)	0.55 (0.66) 492 (95.7) 16 (3.1) 6 (1.2)	0.02 (*p* = 0.616)
Being considerate in road traffic (e.g., respecting speed limits and avoiding high speed)	Mean (SD) Never N (%) Frequently N (%) Always N (%)	3.03 (0.89) 32 (6.2) 91 (17.7) 391 (76.1)	3.01 (0.88) 29 (5.6) 97 (18.9) 388 (75.5)	−0.02 (*p* = 0.643)
Physical activity (ensuring regular exercise)	Mean (SD) Never N (%) Frequently N (%) Always N (%)	2.40 (1.09) 110 (21.4) 175 (34.0) 229 (44.6)	2.37 (1.07) 127 (24.7) 155 (30.2) 232 (45.1)	−0.03 (*p* = 0.460)

* negatively oriented items; “never” included answer options “never” and “rarely”; “frequently” included answer option “frequently,” “always” included answer options “nearly always” and “always.” Hedges’ g = effect size, bias-corrected version of Cohen’s d (Borenstein et al., 2009), classification g ≥ 0.2 as small, g ≥ 0.5 medium, and g ≥ 0.8 large (Cohen, 1988). Effects with type-I-error probability below 0.05 are highlighted in bold.

With the exception of “regular health screening” and “following medical orders,” both of which increased, five of the seven health behaviors decreased (mean increased for inverse items). Significant changes from the baseline (t1) to the follow-up (t2) were observed for AYAs reports of “regular health screening” (Hedges’ g = 0.44), which increased significantly (Table 2).

### Differences Between Low- and Highly Distressed Patients

Group differences between low- and highly distressed patients at t2 were significant for the “being physically active” item, as well as for the nutrition item “keeping an appropriate and balanced diet” (Hedges’ g = −0.21; Hedges’ g = −28) (Table 3).

**TABLE 3:** Health behavior dimensions for low- and highly distressed AYA patients at t2 (*n* = 514).

	**Low distress**	**High distress**	**Difference Hedges’ g**
	***n* = 392 M (SD)**	***n* = 122 M (SD)**	
Regular health screening (e.g., medical check-ups and functional testing of organs)	3.28 (1.07)	3.28 (1.08)	0.00 (*p* = 0.968)
Following medical orders (e.g., medication intake)	3.48 (0.78)	3.34 (0.86)	−0.18 (*p* = 0.084)
Keeping an appropriate and balanced diet (rich in vitamins, minerals, and fiber)*	2.57 (0.91)	2.37 (0.96)	−**0.21 (*p* = 0.039)**
Consumption of nicotine* (smoking cigarettes, cigars, pipe)	0.50 (1.11)	0.63 (1.26)	0.11 (*p* = 0.279)
Consumption of “harder” alcoholic beverages* (liquor, etc.)	0.57 (0.66)	0.51 (0.67)	−0.09 (*p* = 0.377)
Being considerate in road traffic (e.g., respecting speed limits and avoiding high speed)	3.02 (0.89)	2.97 (0.85)	−0.05 (*p* = 0.625)
Physical activity (ensuring regular exercise)	2.44 (1.07)	2.15 (1.04)	−**0.28 (*p* = 0.008)**

*Hedges’ g = effect size, bias-corrected version of Cohen’s d (Borenstein et al., 2009), classification g ≥ 0.2 as small, g ≥ 0.5 medium, and g ≥ 0.8 large (Cohen, 1988). Effects with type-I-error probability below 0.05 are highlighted in bold. Highly distressed patients: patients exceeding the cut-off of ≥ 11 on at least one HADS subscale (anxiety or depression). Low distressed patients: patients with cut-off of < 11 on HADS anxiety or depression subscale.*

### Factors Associated With Health Behavior

In four of the seven models (“regular health screening,” “keeping an appropriate and balanced diet,” “consumption of fatty meals,” and “being considerate in road traffic”), a significant association with gender was identified, with women reporting more health-conscious behavior than men. Higher QoL was associated with more health behavior in three of the seven indicators of health behavior (“keeping an appropriate and balanced diet,” “being physically active,” and “being considerate in road traffic”). More pain was significantly associated with “being considerate in road traffic.” More financial difficulties were significantly associated with lower “consumption of harder alcoholic beverages.” For the independent factors age at diagnosis, fatigue, and distress, no significant association was detectable in any of the models. In summary, the seven regression models explained up to 10% of the health behavior, indicating small effects (Table 4). The largest correlation in terms of magnitude is for QoL and fatigue and is less than 0.8 with | *r*| = −0.68. Multicollinearity between the UVs can be excluded.

**TABLE 4:** Multiple linear regressions/associated factors of health behaviors at t2 (*n* = 514).

**B (*p*-value) *n* = 514**	**Health screening**	**Medical orders**	**Balanced diet**	**Nicotine**	**Harder alcoholic beverages**	**Road traffic**	**Physical activity**
Δ*R*^2^	0.043	0.024	0.051	0.012	0.063	0.083	0.104
ΔF (df), *p*-value	**3.24 (7), 0.002**	1.79 (7), 0.087	**3.88 (7), <0.001**	0.85 (7), 0.547	**4,89 (7), <0.001**	**6.52 (7), <0.001**	**8.41 (7), <0.001**
Cohen’s f^2^	0.04 Small	0.02 Small	0.05 Small	0.01 Negligible	0.06 Small	0.08 Small	0.10 Small
(Intercept)	**2.95 (<0.001)**	**3.34 (<0.001)**	**2.33 (<0.001)**	**2.37 (<0.001)**	**0.69 (<0.001)**	**0.47 (<0.001)**	**2.68 (<0.001)**
Sociodemographic							
Gender	**0.45 (<0.001)**	*0.16 (0.056)*	**0.29 (0.003)**	0.07 (0.587)	**−0.19 (0.006)**	**0.42 (<0.001)**	**−**0.03 (0.790)
**Medical**							
Age at diagnosis^z^	0.04 (0.411)	0.03 (0.335)	*0.07 (0.068)*	**−**0.04 (0.485)	**−0.10 (<0.001)**	0.01 (0.855)	**−**0.06 (0.228)
Pain^1z^	0.07 (0.274)	**−**0.02 (0.735)	0.03 (0.585)	**−**0.01 (0.938)	**−**0.02 (0.545)	**0.17 (<0.001)**	**−**0.09 (0.120)
Fatigue^1z^	0.021 (0.827)	0.02 (0.738)	0.02 (0.751)	0.01 (0.902)	0.07 (0.113)	**−** *0.11 (0.053)*	**−**0.03 (0.682)
**Psychosocial**							
Quality of life^1z^	0.10 (0.165)	0.07 (0.215)	**0.17 (0.007)**	**−**0.02 (0.798)	0.05 (0.255)	**0.14 (0.014)**	**0.28 (<0.001)**
Distress^2^	**−**0.02 (0.851)	**−**0.07 (0.449)	**−**0.12 (0.254)	0.04 (0.752)	0.02 (0.815)	0.07 (0.518)	0.10 (0.426)
Financial difficulties^1z^	0.04 (0.432)	**−**0.04 (0.274)	0.06 (0.189)	*0.10 (0.079)*	**−0.07 (0.030)**	0.00 (0.919)	0.04 (0.370)

*T2 health behaviors were regressed on independent variables at t2. Coding of independent variables: gender: 0 = male, 1 = female; age at diagnosis: measured in years, z-standardized; pain and fatigue: 0–100, high values indicate a higher level of the respective burden, z-standardized; quality of life: 0–100, higher values indicate better quality of life, z-standardized; distress: 0 HADS anxiety and depression scores < 11, 1 at least one score = 11; financial difficulties: 0–100, higher values indicate more difficulties, z-standardized. Abbreviations: B unstandardized regression coefficient; p type-I-error probability; Δ*R*^2^ change in explained variance after including the independent variables; ΔF corresponding change in F-value, df degrees of freedom; independent variables marked superscript ^*z*^ were standardized to mean = 0 and standard deviation = 1; bold: *p* < 0.05, *italic*: *p* < 0.10.*

## Discussion

The aim of this study was to investigate young cancer patients’ health behaviors and to identify associated sociodemographic, medical, and psychosocial factors. Although a healthy lifestyle is essential for improving long-term health outcomes and QoL of young cancer survivors (Pugh et al., 2020), this is the first study on the presence of a range of various health behaviors.

### Health Behavior Indicators

The results indicated that AYAs showed a predominantly health-conscious lifestyle. For example, only one in 10 AYA patients reported smoking frequently or always, and 4% of the patients reported drinking harder alcoholic beverages frequently or always, which is comparable to a recent study by Pugh et al. (2020) in 13–25-year-old cancer patients, where only very few were smokers, but 32% reported alcohol consumption. The same study also included controls: 80.4% of controls reported drinking no more than two alcoholic drinks per day and 86.0% were non-smokers. In their review, Ford et al. (2014) reported that 40–70% of AYA cancer survivors reported good dietary habits. However, a summary by Daniel et al. (2015) of a workshop on the needs and lifestyle challenges of AYA cancer patients found that a substantial proportion of AYAs did not meet the guidelines for healthy eating, including sufficient intake of fruit/vegetables or several nutrients like calcium, vitamin D, folate, and iron and avoiding high fat and calorie intake. This is reflected in our sample with regard to “keeping an appropriate and balanced diet,” with nearly half of the respondents answering never or only frequently. As regards the comparison with the general population, about 75% consume too much fat (Pugh et al., 2020). One in four patients reported never being physically active. Given the effectiveness of physical activity for cancer recovery and general physical health (McTiernan et al., 2019), this is problematic and requires targeted promotion.

### Changes Over Time and Group Differences

With regard to the effect size of changes over time, only “Regular health screening” might be clinically relevant. One explanation could be patients’ growing awareness of the opportunities associated with early health screening, respectively, the fear of a recurrence of cancer. Nevertheless, our findings show a small effect size and need to be investigated in further studies.

There were almost no group differences as a function of psychological distress, which can be viewed as positive, because it means that even highly distressed patients do not necessarily live less healthily than low-distressed patients. Nevertheless, the analysis of low- and highly distressed patients yielded that highly distressed AYAs are less physically active and keep less an appropriate and balanced diet than low-distressed AYAs. Of course, having cancer makes it even harder to maintain an active lifestyle (for example, because of the treatments’ side effects and the long hospital stays). Another important factor which could have an influence on health behavior and which was not surveyed in this study is perceived health status and social support (Thomas and Borrayo, 2011) and further, patients’ health literacy: AYAs can only live healthily if they are educated on living healthily.

Zebrack (2009) revealed that 79% of the surveyed AYAs wished for more information about how to establish and sustain an active lifestyle. Richter et al. (2019) reported that only 28% of the surveyed cancer survivors achieved a sufficient health literacy. Besides a lack of health literacy, there is also a lack of health promoting interventions in general. Demark-Wahnefried et al. (2005) criticize that although promising interventions are available, only 20% of professionals offer such interventions.

### Factors Associated With Health Behavior

Patients’ gender and QoL were associated with health behavior relatively consistently, but mildly rather than strongly. Women reported more health-conscious behavior than men in four out of seven health behaviors. This pattern of health behavior has also been shown in the general population (Berrigan et al., 2003) and does not appear to be an AYA specific effect. If women in the general population and in AYA cancer survivors report more health-conscious behavior than AYA men, it is therefore important to target AYA men in terms of health promotion and offer them specific health promotion programs. We found a relationship between health behaviors and QoL. This relationship could conceivably go either way: better health behaviors lead to better QoL or better QoL is associated with better health behaviors, possibly mediated by other underlying variables.

While there are studies that have found an association between pain and substance consumption (Smith et al., 2017), our findings do not replicate these findings but show associations between higher pain and being more considerate in road traffic. Also, no association was found between fatigue and health behavior, so the results of other studies are not replicated (Spathis et al., 2017).

Surprisingly, psychological distress was no associated with health behavior. This result can perhaps be attributed to the variety of concepts of health behavior and its heterogeneous operationalization in different studies. Moreover, Rabin (2011) pointed out that constant fear (of a recurrence of the disease, for instance) might contribute to an overall healthier lifestyle in young adult cancer survivors. The use of anxiety and depression as indicators of distress, and the lack of an indicator of fear of disease progression, may be another reason why no relationship was found between distress, and health behaviors in this study, but was found between QoL and health behaviors. The unspecific assessment of anxiety and depression—and so an unspecific investigation of fear of progression—might be another reason that no association between distress but QoL and health behavior was found in this study.

After excluding potential influencing factors, it is even more important to explore health behavior further, to disclose its structure, and a way to consolidate it. Motivational, intentional, and volitional processes, as well as social and socio-structural influences, are described as conditions for actual health behavior (Faltermaier, 2015; Beenackers et al., 2018).

### Limitations

One limitation of our study was that we surveyed more young women than men (75% women). Although women between 18 and 39 years old are more often diagnosed with cancer than their male contemporaries (Dong et al., 2020), the imbalance in gender is significantly greater in our sample compared to official cancer registry data (Leuteritz et al., 2018). Possible reasons were discussed for this phenomenon earlier (Leuteritz et al., 2018). Other clinical trials done on cancer patients have also shown that males are less likely to participate and have included samples with a similar 1:4 ratio of men to women (Kaul et al., 2017; Dawson et al., 2018). There is the possibility that having cancer is not a decisive influential factor in respect of health behavior, which might be important for developing and establishing interventions, but there is also the possibility that more health-conscious behavior begins with the diagnosis or treatment. This is shown in the study by Pugh et al. (2020), where AYAs were retrospectively interviewed in this respect. Furthermore, within the framework of the present study, potential associated factors were explored, but there are still many to consider, such as self-efficiency or other comorbid diseases, which could be relevant with regard to health behavior. In addition, we assessed health-related behaviors using survey items rather than domains, which is a limitation on the one hand because individual items with few response options are generally less reliable than scores. On the other hand, however, it is also a strength, as more nuanced conclusions can be drawn than when items are combined into an overall score. Also, considering health behavior as a combination of different behavioral patterns, the present study did not cover all of them: for example, the consumption of illegal substances as a harmful-to-health behavior is not covered. Those which were covered were partially limited to a simple response format that does not provide information about frequency of drinking alcohol and smoking cigarettes. Even though this study is constructed longitudinally, a healthy comparison group, which could help classifying AYAs’ level of health behavior compared to the general population, is missing, along with whether health-oriented interventions need to be specifically adapted for AYAs.

### Implications

Future research should focus on associations between health behavior among AYAs and the individual course of their disease, to estimate the potential cumulative effects of cancer, psychological distress, and reduced health behavior. Moreover, it is necessary to identify factors which are more strongly associated with health behavior to capture the possible impacts and to get a better understanding of the construct of health behavior. Also, as a more clinical implication, the results may indicate possible gaps in care—e.g., considering health education or target-oriented interventions—which might appear as barriers for AYA survivors and their individual health behaviors and need to be approached.

## Data Availability Statement

The raw data supporting the conclusions of this article will be made available by the authors, without undue reservation.

## Ethics Statement

The studies involving human participants were reviewed and approved by the ethics committee of the medical faculty of the University of Leipzig, Germany (Ref.-Nr. 372-13-16122013). The patients/participants provided their written informed consent to participate in this study.

## Author Contributions

KG, MF, and IS: conceptualization of the study. MF and KG: methodology. MF, IS, KG, and KL: formal analysis. IS and KG: investigation and writing – original draft preparation. IS, KG, KL, RS, MF, DR, and AS: writing – review and editing. KG and KL: supervision. AS and KG: funding acquisition. All authors read and approved the final manuscript.

## Conflict of Interest

The authors declare that the research was conducted in the absence of any commercial or financial relationships that could be construed as a potential conflict of interest.

## Publisher’s Note

All claims expressed in this article are solely those of the authors and do not necessarily represent those of their affiliated organizations, or those of the publisher, the editors and the reviewers. Any product that may be evaluated in this article, or claim that may be made by its manufacturer, is not guaranteed or endorsed by the publisher.

## Abbreviations

AYA: adolescents and young adults.
