# Supplementary material for: Health Behavior and Associated Factors in Young Adult Cancer Patients
Source: Front Psychol. 2021 Sep 1;12:697096. doi: 10.3389/fpsyg.2021.697096 (PMC8440830; doi:10.3389/fpsyg.2021.697096)
Supplement: Supplementary file 1 [file Table_1.docx]

**Original Research Article**

**Health behavior and associated factors in young adult cancer patients**

**Isabelle Stroske^1^*, Kristina Geue^1^, Michael Friedrich^1^, Annekathrin Sender^1^, Ricarda Schmidt^2^, Diana Richter^1^, Katja Leuteritz^1^**

^1^Department of Medical Psychology and Medical Sociology, University Medical Center Leipzig, Leipzig, Germany

^2^Clinic and Polyclinic for Psychosomatic Medicine and Psychotherapy, University Medical Center Leipzig, Leipzig, Germany

Supplementary Table S1. Analysis of multicollinearity between the independent variables.

|  | Mean (SD) | | | Correlations (Pearson’s r) of independent variables | | | | | | |
| --- | --- | --- | --- | --- | --- | --- | --- | --- | --- | --- |
|  |  | |  | Gender | Age at Diagnosis^3^ | Pain^3^ | Fatigue^3^ | Quality of Life^3^ | Distress | Financial Difficulties^3^ |
| Gender | 0.75 (0.433) |  | | 1 | 0.124 | 0.170 | 0.158 | -0.097 | 0.089 | 0.052 |
| Age at Diagnosis^3^ | 29.63 (6.14) |  | | 0.124 | 1 | 0.095 | 0.071 | -0.139 | 0.079 | 0.073 |
| Pain^1,3^ | 24.69 (29.02) |  | | 0.170 | 0.095 | 1 | 0.552 | -0.579 | 0.282 | 0.349 |
| Fatigue^1,3^ | 44.00 (28.08) |  | | 0.158 | 0.071 | 0.552 | 1 | -0.683 | 0.429 | 0.358 |
| Quality of Life^1,3^ | 69.18 (19.74) |  | | -0.097 | -0.139 | -0.579 | -0.683 | 1 | -0.475 | -0.373 |
| Distress^2^ | 0.24 (0.426) |  | | 0.089 | 0.079 | 0.282 | 0.429 | -0.475 | 1 | 0.246 |
| Financial Difficulties^1,3^ | 33.47 (37.47) |  | | 0.052 | 0.073 | 0.349 | 0.358 | -0.373 | 0.246 | 1 |
| Notes: ^1^ EORTC-QLQ-C30; ^2^ HADS - Hospital Anxiety and Depression Scale; ^3^ z-standardized before inclusion into the respective model. | | | | | | | | | | |
